# Trend of hospitalized cases of oral cancer in Brazil and its relationship with oral health coverage in public health system between 2009 and 2017

**DOI:** 10.4317/medoral.24009

**Published:** 2020-11-28

**Authors:** Maria Letícia B Raymundo, Aldelany R Freire, Deborah E W Gomes-Freire, Rennis O Silva, Elza C F Araújo, Renato T P Ishigame, Simone A Sousa, Edson H G Lucena, Yuri W Cavalcanti

**Affiliations:** 1 School of Dentistry. Federal University of Paraíba (UFPB), João Pessoa, Brazil; 2 Department of Clinical and Social Dentistry. Federal University of Paraíba (UFPB), João Pessoa, Brazil; 3 General Coordination of Oral Health. Family Health Department. Primary Care Secretary. Ministry of Health. Federal District, Brazil

## Abstract

**Background:**

This study aimed to analyze the trend in the number of hospitalized cases of oral cancer in Brazil, according to the coverage of oral health services in public health system, and also investigate the influence of healthcare and clinical characteristics on the severity of oral cancer cases.

**Material and Methods:**

This retrospective study considered the period between 2009 and 2017. Data from the Hospital Registry of Cancer from the National Cancer Institute were used, considering the primary locations C00 to C06. Detailed information including sex, age, alcohol and tobacco use, year of first consultation, and the clinical stage of the cases were also collected. The frequency of hospitalized cases was correlated with the coverage of Primary Care Oral Health Teams (ESB) and the number of Dental Specialty Centers (CEO). It was also estimated the chance of advanced oral cancer cases, according to healthcare and clinical characteristics. Data were analyzed using Tweedie's multiple regression and multiple binary logistic regression (α<0.05).

**Results:**

There was an increasing trend in the number of hospitalized cases of oral cancer in Brazil between 2009 and 2017 (B=0.043, p<0.001, PR=1.044). The increase in ESB coverage was associated with small increase in the number of hospitalized cases of oral cancer (B=0.001, *p*=0.003, PR=1.001). The increase in the number of CEO was associated with decrease in the number of hospitalized cases of oral cancer (B=-0.085, *p*<0.001, PR =0.918). The increase of ESB (OR=0.998) and CEO (OR=0.974) contributed for reducing the number of stage IV cases, whilst the history of alcohol and tobacco use (OR=1.574) was associated with an increase in the number of stage IV cases.

**Conclusions:**

Although an increasing trend was detected, the expansion of the public health system reduced the number of hospitalized cases and the frequency of advanced oral cancer cases in Brazil.

** Key words:**Mouth neoplasms, squamous cell carcinoma, oral diagnosis.

## Introduction

The incidence of oral cancer in Brazil is considered one of the highest in the world, with high rates of morbidity and mortality, which highlights its relevance in public health (1-3). Oral cancer is among the six most common types of cancer in males and among the eight most prevalent in females (2,3). The most frequent histological type is squamous cell carcinoma (SCC), which consists of about 90% of cases and is routinely diagnosed on the lateral and ventral surfaces of the tongue, floor of the mouth and lower lip (2,3).

The Brazilian National Cancer Institute (INCA) estimated 11,200 new cases of oral cancer in male and 3,500 new cases in female (4), for each year of the 2018-2019 biennium. The epidemiological characteristics of the affected patients are, in their majority, men over 40 years of age, of low socioeconomic status and low educational level (5,6). The main risk factors are smoking and alcohol abuse. In many cases, late diagnosis and difficulty in accessing health services are associated with increased oral cancer morbidity and mortality (7,8).

In Brazil, the universal public health system recently completed 30 years of existence, but the oral health services were included in public health only in the early twenty-first century. The significant expansion of the primary care in poorest regions of the country tried to overcome social inequalities and geographical access to health services, considering the principle of equity (9). In 2000, the Oral Health Teams (ESB) become part of the primary care (10), and four years after this insertion, the Brazilian National Oral Health Policy (Smiling Brazil) was established. Since then, there has been an expansion of the oral health network, especially in primary health care in Brazil.

In addition to establishing the expansion and qualification of oral health in primary health care, the Smiling Brazil program included strategies that stimulated the prevention and control of oral cancer (9). The performance of preventive exams for early detection of cancer in health units was encouraged, in order to identify cases still at an early stage that would allow therapeutic interventions, in an attempt to increase patients' survival and quality of life (11). Smiling Brazil also made it possible to create the Oral Health Specialized Centers (CEO), which places oral diagnosis as one of the minimum specialties to be offered, with an emphasis on the diagnosis and detection of oral cancer (5).

It is possible that the expansion of the offer of oral health services in the Unified Health System (SUS) has influenced the number of hospitalized cases of oral cancer in Brazilian cities. Thus, this study aimed to analyze the trend in the number of hospitalized cases of oral cancer in Brazil, as well as to verify the association with the expansion of oral health coverage in primary care and an increase in the number of oral health specialized centers, from 2009 to 2017. In addition, this study also investigated the influence of healthcare and clinical characteristics on the severity of oral cancer cases.

## Material and Methods

- Study design

We carried out an observational study with retrospective design, considering the number of hospitalized cases of oral cancer in Brazil, between 2009 and 2017. Analyses were carried out both considering the number of hospitalized cases within each city, and the severity of cases diagnosed for each patient.

- Data collection

The number of hospitalized cases of oral cancer in each Brazilian city was obtained from the Hospital Cancer Registry database of the National Cancer Institute (https://irhc.inca.gov.br/RHCNet/visualizaTabNetExterno.action). The number of first consultation records was obtained for each year of study, according to the city of residence, considering the primary locations: lip, base of the tongue, tongue, gums, floor of the mouth, palate, other parts of the mouth not specified (C00 to 06). Only cities that reported at least one case of oral cancer in the period 2009-2017 were included.

In addition, more detailed information for each oral cancer lesion within 2009-2017 was also obtained from the Hospital Cancer Registry database of the National Cancer Institute (https://irhc.inca.gov.br/RHCNet/visualizaTabNetExterno.action). Detailed information included sex, age, alcohol and tobacco use, year of first consultation, and the clinical stage of the cases. Clinical stage was dichotomized into stages I-III and stage IV. Cases without clinical stage definition or with missing information were excluded for analysis.

The population size, the oral health coverage in primary care and the number of oral health specialized centers, by Brazilian city, between the years 2009 and 2017, were extracted from the e-Gestor portal, under the management of the Health Department of Ministry of Health (https://egestorab.saude.gov.br/).

- Data analysis

The data were tabulated and analyzed using the IBM Statistical Package for Social Sciences program (IBM SPSS, v. 24, IBM, Chicago, IL). All analyses were performed considering the significance of 5%.

Tweedie's multiple regression was used to verify the effect of the year, oral health coverage in primary care and the number of oral health specialized centers on the number of hospitalized cases of oral cancer in Brazilian cities. Tweedie multiple regression was used because it allows the analysis of quantitative data, with a distribution similar to the gamma, with multiple true zeros (which mean absence of hospitalized cancer case). Tweedie’s multiple regression model aimed to assess how much the dependent variable can be modified according to variations in independent variables. The beta regression coefficient expresses how much the dependent variable (number of hospitalized cancer cases) is expected to change according to the one-unit increase within the independent variables with *p*<0.05. Values of prevalence ratio (PR) and 95% confidence interval were used to estimate the effect of independent variables on the number of hospitalized cases of oral cancer. Model adjustment was assessed through Omnibus test (*p*<0.05).

**Table 1:**
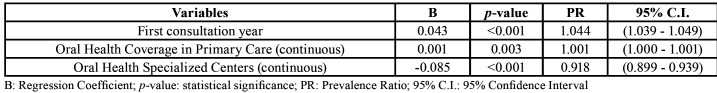
Tweedie multiple regression that verified the effect of the first consultation year, oral health coverage in primary care, and the number of oral health specialized centers in the number of hospitalized cases of oral cancer in Brazilian cities, between 2009 and 2017.

**Table 2:**
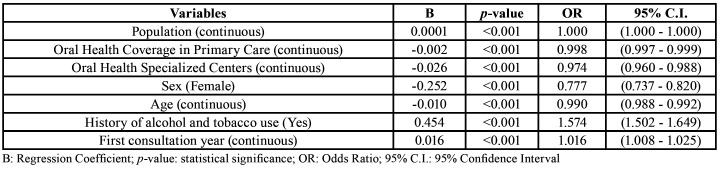
Multiple binary logistic regression used to estimate the effect of healthcare and clinical characteristics on the severity of oral cancer cases in Brazil, between 2009 and 2017. Logistic regression analysis determined the chance of stage IV oral cancer cases.

**Table 3:**
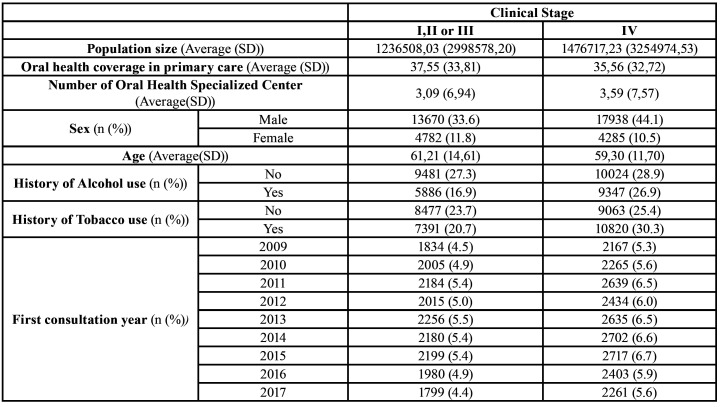
Descriptive data regarding the distribution of healthcare and clinical characteristics according to clinical stage of oral cancer.

In addition, multiple binary logistic regression was used to assess the effect of healthcare and clinical characteristics on the severity of oral cancer cases, using a hierarchical approach, based on the theoretical model proposed by Dahlgren and Whitehead. Healthcare characteristics included population size, primary care coverage, and the number of oral health specialized centers. Clinical characteristics included sex, age, and alcohol and tobacco use, and first consultation year. Initially, only healthcare characteristics were assessed for significant associations. Variables with *p*<0.05 were kept within the model in which clinical characteristics were also tested. Odds ratio (OR) and 95% confidence interval were used to estimate the chance of stage IV oral cancer cases, according to healthcare and clinical characteristics. Model adjustment was assessed through Omnibus test (*p*<0.05).

## Results

Between 2009 and 2017, of the 5570 Brazilian cities, 4,516 were included for analysis. In this period, 72,256 cases were identified. The oral health coverage in primary care varied between 0% and 100%, and the average (sd) per city was 37.42% (43.11%). With regards to the number of oral health specialized centers, the number varied between 0 and 30, and the average (sd) per city was 0.11 (0.53).

Tweedie's multiple regression (Table 1) showed an increasing trend in the number of hospitalized cases of oral cancer in Brazil between 2009 and 2017 (B = 0.043, *p* <0.001, OR = 1.044). The increase in oral health coverage in primary care was associated with an insignificant increase in the number of hospitalized cases of oral cancer (B = 0.001, *p* = 0.003, OR = 1.001). The increase in the number of oral health specialized centers in Brazil was associated with a reduction in the number of hospitalized cases of oral cancer (B = -0.085, *p* <0.001, OR = 0.918).

However, by analyzing the severity of oral cancer cases, we detected that the increase of oral health coverage in primary care (OR = 0.998) and the increase in the number of oral health specialized centers (OR = 0.974) contributed to the reduction on the frequency of stage IV oral cancer cases (Table 2). In addition, clinical characteristics like female sex (OR = 0.990) and greater age (OR = 0.777) were associated with lower chance of stage IV oral cancer cases, whilst history of alcohol and tobacco use (OR = 1.574) was associated with a greater chance of stage IV oral cancer cases. An increasing tendency IV oral cancer cases was detected (OR = 1.016). Descriptive data regarding the distribution of healthcare and clinical characteristics according to clinical stage of oral cancer is presented in Table 3.

## Discussion

The results of the present study indicate a significant increase in registered cases of oral cancer in Brazil, between 2009 and 2017. The increase in the number of cases may be related to the expansion of the oral health services network, that increased the oral health coverage in primary care and led to the expansion and qualification of the specialized care with the implantation of oral health specialized centers, encouraged by the Smiling Brazil program (12).

Although a small increase in the number of cancer cases was detected due to the increase in the coverage of primary care, it is necessary to recognize that the expansion of health services is associated with a decrease in the number of hospital referrals (12,13). In general, the professional active in the oral health primary care service is involved with less complex procedures. Over the decades, there was no improvement in the survival of patients with oral cancer, due to the late search for care (14). In this sense, in the face of a suggestive diagnosis of oral cancer, the preferred conduct of the oral health team in primary care is to refer the patient to a more complex health service.

The present study also showed that the expansion of oral health coverage in primary care and the increase in the number of specialized centers contributed to a decrease in severe hospitalized cases of oral cancer. Based on that, we can infer that the identification of early-stage injuries in the primary care and referral of these patients to oral health specialized centers can contribute to increasing the rate of resolution of cases and reducing the hospital demand of patients with advanced-stage injuries (15).

This investigation demonstrated an increasing trend in advanced cases of oral cancer. Thus, as the perspective of strengthening primary care and the public health system, as well as reducing the number of hospitalized and severe cases, a focus on preventive work is recommended. The expansion of oral health care network can certainly have an impact in combating risk factors, which can be addressed by the dental surgeon during routine consultations, as well as increase the frequency of early diagnosis of premalignant lesions (16).

With regard to the control of risk factors, it is necessary to consider the contribution of socioeconomic parameters. According to the International Head and Neck Cancer Epidemiology Consortium (INHANCE), socioeconomic status is considered a risk factor for head and neck cancer, even when controlled by other variables, as well as the concomitant use of alcohol and tobacco, which imply a higher prevalence of advanced stage of oral cancer (13). Socioeconomic inequality also influences cancer patient survival, as it directly reflects limited access to diagnosis and, consequently, treatment (17). The 5-year survival rates are higher in richer countries than in lower and middle-income countries. These data demonstrate great disparities from diagnosis to cancer treatment, reflecting, in part, differences related to investment in cancer control in countries around the world (18).

The diagnosis of oral cancer, involving biopsy and exfoliative cytology, as well as other complementary exams, is optional for primary care dentists, and ideally, everyone should be trained to collect, order and interpret the results (19). Individuals diagnosed with oral cancer have a higher frequency of clinical stage IV (13,16), due to the late search for care, considering that initial injuries, in most cases, have no symptoms, being underestimated by the individual himself and by health professionals (15). Among these individuals, this study shows that men are the majority affected by stage IV cancer, as well as those who abuse of alcohol and tobacco, corroborating previous findings in the literature (1,8,13).

Sometimes, there is little evidence of resolvability of the primary care in public health services. Constant referral to specialized centers leads the patient to a hegemonic logic strongly based on the hospital-specialist-disease (19,20). There is a need for improvements in the structure and, consequently, in the assistance provided to the patient affected by oral cancer who seeks primary care (12,20). Based on our findings, the association between the increase in the number of oral health specialized centers and the reduction of the number of hospitalized (and severe) cases of oral cancer in Brazil, may mean that oral health specialized attention has contributed to the increase in resolvability. These services act in a complementary way to the care offered in primary care, so the prevention and diagnosis of oral malignancies, at the appropriate time, are the most effective measures of cancer prognosis (15). Future studies need to assess the effect of expanding the health care network on increased survival and reduced mortality from oral cancer.

This study has some limitations, as it is an analysis of secondary data, focused on hospital cancer records, which should not be used to estimate the risk of cancer within the population. Nevertheless, the INCA database is considered the most solid source of cancer data in Brazil. It must be considered, however, that the results of this study are representative for Brazil and comprise an analysis of the last decade of hospital oral cancer records. The findings of this study are useful to justify the need for expansion and greater investment in the Brazilian public health system.

## Conclusions

The number of hospitalized cases of oral cancer registered in hospitals in Brazil showed an increasing trend between the years 2009 and 2017. It was found that the expansion of the oral public health service network, especially represented by the increase in the number of oral health specialized centers, contributed to reducing the frequency of hospitalized cases of oral cancer. The severity of hospitalized oral cancer cases was negatively associated with the expansion of the oral public health service network, and positively with males and the consumption of tobacco and alcohol. These findings demonstrate the importance of building the oral public health network to reduce health inequalities.
